# Usefulness and safety of 0.4% sodium hyaluronate solution as a submucosal fluid "cushion" for endoscopic resection of colorectal mucosal neoplasms: A prospective multi-center open-label trial

**DOI:** 10.1186/1471-230X-9-1

**Published:** 2009-01-08

**Authors:** Shoji Hirasaki, Takahiro Kozu, Hironori Yamamoto, Yasushi Sano, Naohisa Yahagi, Tsuneo Oyama, Tadakazu Shimoda, Kentaro Sugano, Hisao Tajiri, Takao Takekoshi, Daizo Saito

**Affiliations:** 1 Department of Internal Medicine, Sumitomo Besshi Hospital, Ehime, Japan; 2 Endoscopy Division, National Cancer Center Hospital, Tokyo, Japan; 3 Department of Internal medicine, Division of Gastroenterology, Jichi Medical University, Tochigi, Japan; 4 Division of Digestive Endoscopy, National Cancer Center Hospital East, Chiba, Japan; 5 Department of Gastroenterology, Toranomon Hospital, Tokyo, Japan; 6 Department of Gastroenterology, Saku General Hospital, Nagano, Japan; 7 Clinical Laboratory Division, National Cancer Center Hospital, Tokyo, Japan; 8 Department of Internal Medicine, The Jikei University School of Medicine, Tokyo, Japan; 9 Department of Gastroenterology, Maeda Hospital, Tokyo, Japan; 10 Nihonbashi Daizo Clinic, Tokyo, Japan

## Abstract

**Background:**

Sodium hyaluronate (SH) solution has been used for submucosal injection in endoscopic resection to create a long-lasting submucosal fluid "cushion". Recently, we proved the usefulness and safety of 0.4% SH solution in endoscopic resection for gastric mucosal tumors. To evaluate the usefulness of 0.4% SH as a submucosal injection solution for colorectal endoscopic resection, we conducted an open-label clinical trial on six referral hospitals in Japan.

**Methods:**

A prospective multi-center open-label study was designed. A total of 41 patients with 5–20 mm neoplastic lesions localized in the colorectal mucosa at six referral hospitals in Japan in a single year period from December 2002 to November 2003 were enrolled and underwent endoscopic resection with SH. The usefulness of 0.4% SH was assessed by the *en bloc *complete resection and the formation and maintenance of mucosal lesion-lifting during endoscopic resection. Safety was evaluated by analyzing adverse events during the study period.

**Results:**

The usefulness rate was high (82.5%; 33/40). The following secondary outcome measures were noted: 1) steepness of mucosal lesion-lifting, 75.0% (30/40); 2) intraoperative complications, 10.0% (4/40); 3) time required for mucosal resection, 6.7 min; 4) volume of submucosal injection, 6.8 mL and 5) ease of mucosal resection, 87.5% (35/40). Two adverse events of bleeding potentially related to 0.4% SH were reported.

**Conclusion:**

Using 0.4% SH solution enabled sufficient lifting of a colorectal intramucosal lesion during endoscopic resection, reducing the need for additional injections and the risk of perforation. Therefore, 0.4% SH may contribute to the reduction of complications and serve as a promising submucosal injection solution due to its potentially superior safety in comparison to normal saline solution.

## Background

The incidence of colorectal neoplasm in Japan is high, and the number of patients with colorectal neoplasm is steadily increasing. The chances of treating colorectal mucosal lesion with endoscopy have increased [1-3]. Depending on techniques and equipment, endoscopic resection of the colon includes polypectomy, endoscopic mucosal resection (EMR) and endoscopic submucosal dissection (ESD) in the colon. Focusing on the physiochemical properties of sodium hyaluronate (SH), Yamamoto *et al. *developed a technique known as EMR using sodium hyaluronate (EMRSH), and reported that when SH was injected into the submucosal layer during EMR, mucosal lesions could be adequately lifted for a sufficient duration to allow safe, reliable, and complete resection without SH diffusion or absorption by the submucosal layer [4,5]. Recently, we proved the usefulness and safety of 0.4% SH solution in endoscopic resection for gastric mucosal tumors [6]. To evaluate the usefulness of 0.4% SH as a submucosal injection solution for colorectal endoscopic resection, we conducted an open-label clinical trial on six referral hospitals in Japan.

## Methods

### Patient selection

The target population of the study was consecutive patients who visited six referral hospitals in Japan from December 2002 to November 2003 on an outpatient basis for colorectal mucosal neoplasms that could be treated endoscopically. All patients underwent confirmation colonoscopy within 4 weeks before endoscopic resection. If a patient had already undergone colonoscopy within 4 weeks before informed consent, the data for that colonoscopy was used as part of the study data to avoid another colonoscopy, thereby minimizing stressful procedures for the patient. The present study was explained to those meeting the following criteria, and informed consent was obtained from 44 patients. Three patients were excluded for meeting the exclusion criteria and the remaining 41 patients were enrolled.

Inclusion criteria consisted of the following: a neoplastic lesion 5–20 mm in diameter localized to the colorectal mucosa; indication for endoscopic resection; and patient age between 20 and 80 years. We limited the size of the neoplasms because the guidelines for the Japanese Society for Cancer of the Colon and Rectum [7] state that neoplasms of ≤ 20 mm in diameter can be completely resected *en bloc*.

Exclusion criteria included: 1) residual or recurrent lesion, 2) lesion accompanied by ulceration, 3) submucosal invasion, 4) pacemaker, 5) advanced malignant neoplasm, 6) history of hypersensitivity to SH, 7) systemic administration of an anticancer agent, 8) severe liver, kidney or cardiovascular disease; 9) pregnancy or lactation, or women who wished to become pregnant during the study and 10) patients judged to be inappropriate for inclusion by a physician.

The present study was conducted in compliance with the Helsinki Declaration and Good Clinical Practice (GCP). The institutional review boards (IRB) of all of the participating institutions (the IRB of Shikoku Cancer Center, the IRB of National Cancer Center Hospital, the IRB of Jichi Medical University, the IRB of Tokyo University, and the IRB of Saku General Hospital) approved all study protocols. Written informed consent was obtained from all patients.

### Concomitant medication

For 7 days before and after endoscopic resection, the use of anticoagulants and antiplatelets that can induce bleeding during endoscopic resection or from the surgical wound, or drugs contraindicated in the treatment of peptic ulcer, were prohibited. During endoscopic resection, the submucosal injection of agents that could affect the assessment of 0.4% SH, such as saline, 3.7% hypertonic saline, 50% glucose and 10% glycerol were prohibited. Except for epinephrine and indigo carmine, nothing was added to submucosal injection solutions. However, the concentrations for epinephrine and indigo carmine were not stipulated as a part of the protocol. Furthermore, medication was allowed to treat coexisting disorders not included in the above and which occurred ≥ 4 weeks before endoscopic resection as well as adverse events that occurred during the study.

### Study device

A solution of 0.4% SH was prepared by dissolving SH (manufactured by Seikagaku Corp, Tokyo, Japan) to a concentration of 0.4% (w/v), the solution was then placed in a 20 mL glass vial and stored at room temperature. The upper limit of 0.4% SH submucosal injection was 40 mL, which is one-tenth of the nontoxic level with intraperitoneal administration, and an appropriate amount was used in each patient.

### Study design

The present study involved six referral hospitals in Japan. Patients who satisfied the inclusion criteria underwent endoscopic resection. The primary outcome measure was assessed by comprehensively evaluating *en bloc *complete resection (*en bloc *resection with a histopathologically negative resection margin) and the lifting and maintaining of a mucosal lesion during endoscopic resection (as the number of additional injections required due to loss of mucosal lesion-lifting) (Table 1). Usefulness rate was defined as the percentage of *en bloc *complete resections that required an additional injection number of 0 or 1. Secondary outcome measures included 1) steepness to which the lesion could be lifted using the solution injected into the submucosal layer (steep: the lesion is elevated vertically toward the lumen side to form a tall bulge, mild: the lesion is dispersed laterally to form a shallow bulge, non-lifted, not evaluable), 2) presence or absence of bleeding, perforation and other intraoperative accidental events, 3) time required for mucosal resection, 4) submucosal injection volume, and 5) ease of mucosal resection using submucosal injection (excellent, good, moderate, poor). An adequately lifted lesion refers to a highly lifted and protruding lesion that appears to push the mucosa vertically into the lumen. In relation to the average difficulty of a typical endoscopic resection (moderate), the ease of mucosal resection was assessed in terms of the extent to which the procedure was simplified using 0.4% SH compared with the conventional technique using normal saline. Safety was analyzed in all patients who underwent endoscopic resection by recording all adverse events that were assessed as having potential causal relationships. In addition, we defined serious adverse events as those events that led to: death, life-threatening conditions, notable disability, prolonged hospital stay, or a requirement of hospitalization for therapy. The patients were followed until adverse events either dissipated or returned to pre-endoscopic resection levels. The feasibility of 0.4% SH performance was assessed based on usefulness and safety. Other investigated items included biopsy diagnosis, pre- and postoperative endoscopic findings, histopathological diagnosis, clinical laboratory tests, and vital signs. These items were assessed as specified in the study protocol. Taking into account the time for wound healing following endoscopic resection, the study period was 8 weeks during which recurrence, residual neoplastic tissue and wound healing at the site of resection were ascertained [8,9]. In addition, although endoscopic resection methods can be classified as EMR with mucosal incision, conventional EMR and ESD, the choice of approach was left to the discretion of the physicians at each facility. Specifically, procedures involving snare resection of the mucosal layer were classified as conventional EMR, those involving snare resection following mucosal incision were classified as EMR with mucosal incision, and those involving dissection of the submucosal layer following mucosal incision were classified as ESD. ESD uses devices such as an insulation-tipped electrosurgical (IT) knife [10], hook knife [11], flex knife [12,13] or needle knife in combination with a small-caliber tip transparent hood (ST hood) [14,15].

**Table 1:** Primary outcome measure

Complete en bloc resection	Complete	Complete	Complete	Incomplete or Not evaluable
Additional count	0	1	2≤	-
Total evaluation^†^	Excellent	Good	Moderate	Poor

† Total evaluation of primary outcome measure was assessed by comprehensively evaluating complete *en bloc *resection and the number of additional injections during endoscopic resection. The primary outcome measure (usefulness rate) was defined as the percentage of excellent or good outcomes according to the criteria noted above.

### Statistical analysis and outcomes

As a general rule, the full analysis set (FAS) comprised all patients who underwent endoscopic resection. All analyses were performed using SAS software (SAS Institute, Cary, North Carolina, USA). Intergroup comparisons were conducted as follows.

Usefulness and safety were described by calculating 95% confidence intervals using the binomial method. Values of p < 0.05 were considered statistically significant. The Wilcoxon two-sample test for continuous outcomes and Fisher's exact test for binary outcomes were used. For clinical laboratory test findings, the McNemar test was used to assess changes before and after endoscopic resection.

### Sample size

Endoscopic resection was clinically assessed based on *en bloc *complete resection as determined by histopathological analysis of the neoplastic lesions. The reported rate of *en bloc *complete resection with normal saline as the submucosal injection solution is 72.9–85% [16-18]. From this, when the rate of *en bloc *complete resection for the 0.4% SH was set at 90%, a sample size of 35 patients was required in order to meet the hypothesized rate of 90% resection with a 95% confidence interval of the length ± 10%. The established target number of patients for 0.4% SH, allowing for potential dropouts, was 42.

## Results

**Figure 1:**
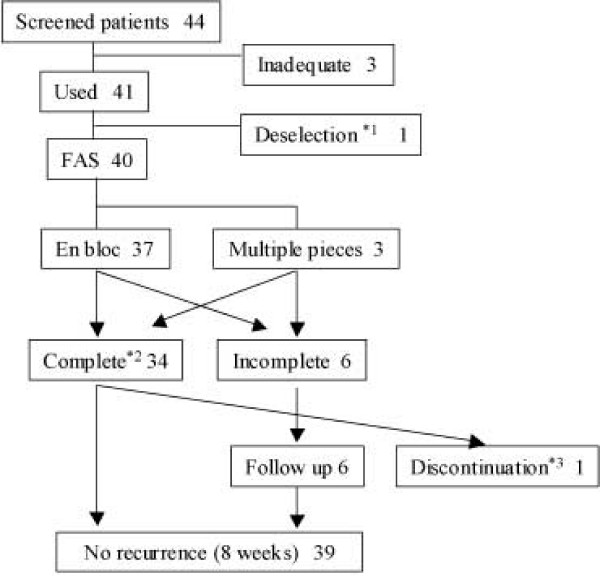
**Trial profile**. Informed consent was obtained from 44 patients. Two patients withdrew informed consent before endoscopic resection. One patient did not have a target neoplasm that was defined in our clinical protocol. Therefore, the final number of patients who underwent endoscopic resection using 0.4% sodium hyaluronate (SH) was 41. In addition, regarding the histopathology of the multifragment resection, if the histopathological judgment was "vertical and lateral negative margins'', then resection was considered complete. *1: One patient met the exclusion criteria. *2: *En bloc *resection with histopathologically negative resection margin. *3: A patient had another neoplasm that was in need of treatment.

The FAS comprised 40 patients, because one patient met the exclusion criteria. We analyzed the safety in all 41 patients. Fourteen patients (34.1%: 14/41) had 0.2 mL–1.0 mL indigo carmine added to 0.4% SH. Fourteen patients (34.1%: 14/41) had 0.1 mL–1.0 mL epinephrine added to 0.4% SH. The final concentration of SH was from 0.38% to 0.4%. Figure 1 shows the flowchart for the study. Six patients were judged as having an incomplete resection. Two patients did not have safe vertical and lateral margin because of multifragment resections (although one additional patient had a multifragment resection, the patient was judged to have had a complete resection because all of the neoplasm was contained in one fragment). In the other four patients, the margin of the fragment was positive for tumor or the lateral margin of the lesion could not be evaluated histologically because of the effects of the electrosurgical current or mechanical damage. Demographic characteristics of the 40 patients at baseline did not reveal differences in age, sex, histological type, or neoplasm size (Table 2).

**Table 2:** Demographic characteristics of the 40 patients at baseline

Characteristic (n = 40)	
Age (years)	
Median	64.0
Range	45 – 80
	
Sex [n (%)]	
Male	27 (67.5)
Female	13 (32.5)
	
Location of neoplasm [n (%)]	
Cecum	2 (5.0)
Ascending colon	7 (17.5)
Transverse colon	10 (25.0)
Descending colon	0 (0.0)
Sigmoid colon	14 (35.0)
Rectum	7 (17.5)
	
Macroscopic classification ^† ^[n (%)]	
I p	5 (12.5)
I sp	10 (25.0)
I s	4 (10.0)
II a	12 (30.0)
Laterally spreading tumor	8 (20.0)
Other	1 (2.5)
	
Neoplasm size [n (%)]	
5–10 mm	19 (47.5)
11–15 mm	13 (32.5)
16–20 mm	8 (20.0)

† In this trial, we classified neoplasms by the General Rules for Clinical and Pathological Studies on Cancer of the Colon, Rectum and Anus edited by the Japanese Society for Cancer of the Colon and Rectum. This macroscopic classification defines the following as the sub-superficial types. Ip: pedunculated type; Isp: semipedunculated type; Is: sessile type; IIa: superficial elevated type.

Conventional EMR comprised most of the endoscopic resections (77.5%; 31/40) performed in the present study (Table 3). Histological examination confirmed that all resected tumors were intramucosal. Adenoma was diagnosed in 32 of the 40 patients (80%).

**Table 3:** Treatment methods and the results on histopathological diagnosis

Patients (n = 40)	
Treatment methods^†^	
Conventional EMR	31 (77.5)
EMR with mucosal incision	3 (7.5)
ESD	6 (15.0)
	
Depth of invasion	
mucosa	40 (100)
submucosa	0 (0.0)
	
Histological type	
adenoma	32 (80.0)
adenocarcinoma	
well differentiated adenocarcinoma (wel)	6 (15.0)
moderately differentiated adenocarcinoma (mod)	0 (0.0)
poorly differentiated adenocarcinoma (por)	0 (0.0)
other^‡^	2 (5.0)

† Conventional endoscopic mucosal resection (EMR) only to snare lesion. EMR with mucosal incision is performed to incise the mucosa and snare the lesion. Endoscopic submucosal dissection (ESD) is performed to incise the mucosa and dissect submucosa.

‡ One patient had a juvenile polyp; the other had a hyperplastic polyp.

### Clinical usefulness

Table 4 shows the assessment results. The primary outcome measure was analyzed by comprehensively evaluating *en bloc *complete resection and additional injections due to a loss in mucosal lesion-lifting (Table 1). The usefulness rate for the 0.4% SH was 82.5% (33/40). The rate of endoscopic resection completion was favorable at all institutions. Only one patient required an additional injection due to the loss of mucosal lesion-lifting; none of the other cases (39/40) required additional injections (Table 4). The mean (SD) of additional injections was 0.1 (± 0.3). We evaluated secondary outcome measures as follows. Mucosal lesion-lifting using 0.4% SH was steep in 75% of 40 patients and the frequency of bleeding, perforation, and other intraoperative accidental events was only 10%. Mild bleeding was encountered in three patients, and perforation occurred in one. Mucosal resection required 6.7 (± 14.3) min for completion. The volume of 0.4% SH was 6.8 (± 8.1) mL and evaluation for ease of endoscopic resection with 0.4% SH revealed excellent or good results in 87.5% (35/40). Each of these five parameters suggests that 0.4% SH simplifies the complex procedures of colorectal endoscopic resection.

**Table 4:** Summary of results

Patients (n = 40)	
Primary outcome measure	
Usefulness rate % (n)	82.5^†^(33)
Complete en bloc resection	82.5 (33)
Additional counts of injection	
none	97.5 (39)
1	0.0 (0)
2	2.5 (1)
	
Secondary outcome measures	
Steepness of mucosal lesion-lifting % (n)	
steep	75.0 (30)
mild	22.5 (9)
non-lifted	0.0 (0)
not evaluable	2.5 (1)
Intraoperative accidental events % (n)	10.0 (4)
Ease of mucosal resection % (n)	
excellent	62.5 (25)
good	25.0 (10)
moderate	5.0 (2)
poor	7.5 (3)
Time required for mucosal resection (min)	
Mean (SD)	6.7 (14.3)
Injection volume (mL)	
Mean (SD)	6.8 (8.1)

† 95% confidence interval: 67.2–92.7

Table 5 shows the relationship between usefulness rate (primary outcome measure) and neoplasm size (as diameter measured endoscopically). Usefulness rate was 89.5% (17/19), for neoplasms of 5–10 mm; 76.9% (10/13), for those of 11–15 mm, and 75.0% (6/8) for those of 16–20 mm. Hence, the usefulness rate was high for 0.4% SH irrespective of neoplasm size. Table 5 also shows the relationship between usefulness rate and neoplasm location. The usefulness rates for 0.4% SH were 0% (0/2) in the cecum, 100% (7/7) in the ascending colon, 80.0% (8/10), in the transverse colon, 92.9% (13/14) in the sigmoid colon and 71.4% (5/7), in the rectum. Again, the usefulness rate was high for 0.4% SH, irrespective of neoplasm location.

**Table 5:** Usefulness rate for neoplasm size and location

Size		
≤ 10	89.5%	(17/19)
11–15	76.9%	(10/13)
16–20	75.0%	(6/8)
		
Tumor location		
Cecum	0%	(0/2)
Ascending colon	100%	(7/7)
Transverse colon	80.0%	(8/10)
Sigmoid colon	92.9%	(13/14)
Rectum	71.4%	(5/7)

Usefulness rate was defined as the percentage of *en bloc *complete resection that required one additional injection or none.

### Adverse events

Adverse events developed in 19 (46.3%) of the 41 patients who underwent endoscopic resection with 0.4% SH. Only bleeding in two patients (4.9%) was judged to be potentially related to the use of 0.4% SH. Colorectal bleeding 1 day after endoscopic resection at the same site was resolved completely within the date of onset in the first patient. In the other patient, the symptom disappeared the next day from the onset and bleeding did not recur thereafter. One patient had a perforation, but it was judged to be unrelated to 0.4% SH. This perforation occurred accidentally during the management of hemostasis after endoscopic resection and was resolved by endoscopic treatment involving only clipping. This perforation was not caused by injection solution or endoscopic resection. All other adverse events were judged to be unrelated to submucosal injection solutions (Table 6). The frequency of serious adverse events, those that prolonged hospital stay or required hospitalization during therapy was 9.8% (4/41). These events were two cases of bleeding, one case of abdominal pain, and one case of perforation (described above). No events led to death, life-threatening conditions or notable disabilities. No patient discontinued this study due to adverse events.

**Table 6:** Adverse events

All Patients (n = 41)	patients		
Patients with adverse events	19	[4]	(2)

Adverse events occurring in ≥ 2 patients^†^			
Abdominal pain	4	[1]	(0)
Post procedural haemorrhage^‡^	4	[2]	(2)
Haemorrhage during procedure^‡^	3	[0]	(0)
Vomiting	2	[0]	(0)
Diarrhoea	2	[0]	(0)
Insomnia	2	[0]	(0)
Intestinal perforation	1	[1]	(0)

All patients who had used 0.4% sodium hyaluronate (SH) were analyzed for adverse events. The square bracket shows the number of patients with serious adverse events. The parenthesis shows the number of patients with adverse events that were judged to be related to 0.4% SH.

† Although one patient experienced intestinal perforation, this event was defined to be a serious adverse event. Therefore, it was added in this table.

‡ Bleeding was defined as the use of clipping or another treatment to stop or prevent bleeding as well as the incidence of actual bleeding.

### Laboratory investigations and vital signs

Total protein, total bilirubin, and blood urea nitrogen levels did not significantly change between the time before endoscopic resection and 1 or 7 days after endoscopic resection using 0.4% SH. No abnormal changes in clinical laboratory findings were correlated with 0.4% SH. No abnormal changes in vital signs were seen in any of the patients.

### Endoscopy

At 8 weeks after endoscopic resection, residual neoplastic tissues or recurrent neoplasms were not evident in any of the patients. The rate of healing (complete loss of white patches at the site of resection at 8 weeks after endoscopic resection) associated with 0.4% SH was 100% (40/40).

### Safety and usefulness

Usefulness (primary outcome measure) was assessed in terms of *en bloc *complete resection and requirement for 0–1 additional injection, and resection was considered safe if no adverse events were determined to be related to submucosal injections and no events led to notable disability or the requirement of additional treatment. Comprehensive assessment of usefulness and safety revealed that the usefulness rate with a 95% confidence interval for 0.4% SH was 82.5% (33/40; 67.2–92.7%).

## Discussion

In colorectal EMR, endoscopists have to acquire the skills of endoscopic technique, submucosal injection, and snaring because the large intestine has anatomical curves and many plicae. Endoscopists often could not adequately lift lesions and maintain those lifted lesions during conventional colorectal EMR using saline, which led to additional injections, failed snaring and/or perforation [19,20]. Incomplete resection leads to residual or recurrent tumors. Complications of EMR such as bleeding and perforation often result in a longer hospital stay and increased hospital costs. Therefore, the mucosal lesion-lifting method allows easy removal of tumors during endoscopic resection, leads to *en bloc *resection, and decreases complications. Some experimental studies [19,21] and case reports [13] have described the effectiveness of SH for EMR. SH is a macromolecular polysaccharide composed of D-glucuronate and N-acetyl-D-glucosamine; it is ubiquitous in human connective tissues and body fluids. SH is physicochemically very water retentive and viscoelastic, and is thus clinically used as a safe intraarticular injection preparation (ARTZ and ARTZ Dispo, Seikagaku, Tokyo, Japan) or as auxiliary compound in cataract surgery (OPEGAN 1.1 and OPEGAN Hi, Seikagaku). Because SH is highly viscoelastic even at concentrations of ≤ 1%, it does not increase osmotic pressure and it is not histotoxic [4,19]. Hyun *et al *described that mucosal elevation lasted longer with 0.1% SH than with normal saline in their study using fresh mongrel transverse colon, and this seemed to be due to the viscosity of the SH solution [20]. However, no multicenter studies have prospectively investigated the usefulness and safety of 0.4% SH as a submucosal injection solution in EMR for colorectal tumors.

We selected 0.4% SH in this study, because Onaya *et al *[22] reported that 0.4% SH created higher protrusion than physiological saline, 50% dextrose, hypertonic saline (3.7% NaCl), and glycerol. In addition, histological analysis showed no tissue injury caused by hypertonicity after 0.4% SH use [22]. In this study, we allowed epinephrine and indigo carmine to be added to submucosal injection solutions. We did not investigate the potential effect of the volumes of indigo carmine and epinephrine because we used such small amounts of these solutions. Therefore, we believe that additional indigo carmine and epinephrine had an insignificant effect on the final concentration of SH.

The intention of calculating a sample size of 35 patients was to keep the length (precision) of the confidence interval to ± 10% from the success rate when the anticipated *en bloc *resection rate was assumed to be 90%. The *en bloc *resection rate for the current study was 92.5% (37/40), and this rate was consistent with our assumption (Figure 1). The *en bloc *complete resection rate was high (82.5%) in this study. In 33 (82.5%) patients, submucosal injection of 0.4% SH was judged to be useful. We had already reported a prospective multicenter randomized controlled study describing that the usefulness rate of 0.4% SH for treating gastric tumors was significantly higher for the 0.4% SH group (88.4%; 61/69) than for the control group (58.6%; 41/70) (p < 0.001) [6]. The usefulness rate of this study of colorectal tumors was nearly as high as that of the gastric tumor study. Uraoka *et al *reported the *en bloc *complete resection rate of 62.0% using glycerol and 34.8% using saline for laterally spreading tumors with size less than 20 mm [23]. In light of such reported findings, we believe that the complete *en bloc *resection rate of 82.5% found in this study is fairly good from a clinical point of view. Although the cost of 0.4% SH is higher than that of saline or glycerol, using 0.4% SH in colorectal endoscopic resection may lead to a higher *en bloc *complete resection rate. In cases of complete resection, patients are generally followed up on a yearly basis. In contrast, incomplete resection leads to residual or recurrent tumor and patients need follow-up endoscopy several times in a year. Thus, incomplete resection leads to increased medical expenses. Using SH in endoscopic resection can be expected to decrease medical expenses for patients and healthcare systems indirectly by potentially reducing the number of hospital visits and follow-up cost.

A steep lift was judged to be adequate in 75.0% of 40 patients in this study and 97.5% of 40 patients required no additional injections for endoscopic resection. No perforation occurred due to endoscopic resection in this study, although one perforation occurred during the management of bleeding. A solution of 0.4% SH, which is highly viscoelastic, provided sufficient formation and maintenance of lifted lesions, prevented perforation and facilitated safer and more complete procedures. These findings lead us to believe that 0.4% SH is useful for the resection of colorectal mucosal lesions.

Adverse events appeared to be at a relatively high rate (46.3%) in this study because we collected all minor adverse events in strict compliance with GCP. Major complications were bleeding during endoscopic resection (7.3% (3/41)) and postoperative bleeding (9.7% (4/41)) (Table 6). The adverse event rates in our study are only slightly higher than the adverse event rates reported by Uraoka *et al *[23] regarding the bleeding during endoscopic resection (0.9% for glycerol and 7.0% for saline) and the postoperative bleeding (6.4% for glycerol and 4.4% for saline). The slightly elevated bleeding rates in our study are thought to be unrelated to the use of SH and to be comparable to the bleeding rates reported in Uraoka *et al*.'s study using glycerol and saline [23]. Nonetheless, it should be kept in mind that because unexpected bleeding may occur during colorectal endoscopic resection and such bleeding is very hard to predict in advance, one should pay careful attention to the possibility of bleeding during endoscopic resection using 0.4% SH as well as other injection solutions.

It must be noted that all procedures in this study were conducted by highly skilled endoscopists familiar with colorectal endoscopic resection whereas in other clinical settings, inexperienced endoscopists might perform colorectal endoscopic resections. Insufficient formation and the inability to maintain lifted lesions seemed to increase the difficulties associated with procedures. A solution of 0.4% SH could adequately lift and maintain lesions regardless of lesion location and the risk of perforation was reduced by creating sufficient space between the muscle layer and the mucosal lesion. In addition, due to its high molecular weight, 0.4% SH is sufficiently viscoelastic at low concentrations, does not affect osmotic pressure and is not associated with tissue damage. These properties may confer advantages as a local injection solution in colorectal endoscopic resection.

This study is a prospective open-label study. Although the study had only a single-arm, a modest sample size and some subjective measurements as potential limitations, the findings of the current study shed light on the usefulness of 0.4% SH as an aid for surgery when compared with available historical control data and reported findings from studies making use of other agents as surgical aids.

## Conclusion

The outcomes in this study suggest that 0.4% SH, which can lift and maintain lesions during resection, can be applied to colorectal endoscopic resection for enhancing the ease and safety of the procedures. The potentially superior safety profile of 0.4% SH supports its use as a promising submucosal injection solution in place of conventional saline solution.

## Abbreviations

GCP: Good Clinical Practice; EMR: Endoscopic mucosal resection; ESD: Endoscopic submucosal dissection; FAS: Full analysis set; IRB: Institutional review boards; IT knife: Insulation-tipped electrosurgical knife; ST hood: Small-caliber tip transparent hood; SH: Sodium hyaluronate

## Competing interests

Seikagaku Corporation, Tokyo, Japan, supported this clinical study to receive approval for the manufacture of a medical device from the Ministry of Health, Labor and Welfare of Japan. Seikagaku Corporation contracted with and paid all hospitals based on GCP as a clinical trial.

HY holds a patent entitled "Method of endoscopic mucosal resection using mucopolysacharide and local injection preparation" filed on May 16, 2000 (United States Patent number 6,319,260).

The other authors had no conflicts of interest regarding this study.

## Authors' contributions

SH participated in this clinical trial as the principal investigator and drafted the manuscript. TK, HY, YS, NY and TO participated in this clinical trial as principal investigators. TS participated on the central pathological judging committee. KS and HT participated in this trial as the safety review board. TT participated in this trial as the medical officer. DS participated in the coordination of this trial and coordination of this manuscript.

## Pre-publication history

The pre-publication history for this paper can be accessed here:

http://www.biomedcentral.com/1471-230X/9/1/prepub
